# Effectiveness of a Multimodal, Day Clinic Group-Based Treatment Program for Trauma-Related Disorders: Differential Therapy Outcome for Complex PTSD vs. Non-Complex Trauma-Related Disorders

**DOI:** 10.3389/fpsyt.2019.00800

**Published:** 2019-11-07

**Authors:** Anke Philipps, Andrea Silbermann, Eva Morawa, Mark Stemmler, Yesim Erim

**Affiliations:** ^1^Department of Psychosomatic Medicine and Psychotherapy, University Hospital of Erlangen, Friedrich-Alexander University Erlangen-Nürnberg (FAU), Erlangen, Germany; ^2^Institute of Psychology, Friedrich-Alexander University Erlangen-Nürnberg (FAU), Erlangen, Germany

**Keywords:** trauma, posttraumatic stress disorder, complex PTSD, therapy outcome, day clinic treatment, posttraumatic growth, social support, depression

## Abstract

**Background:** The effectiveness of the psychotherapeutic treatment of posttraumatic stress disorder is evidence-based and generally considered proven. However, the effectiveness of multimodal, group-based day clinic treatment programs has rarely been investigated. Moreover, there is no consensus in the literature concerning the question whether psychotherapeutic approaches for trauma-related disorders are also applicable for patients with complex PTSD (cPTSD). The aim of the study was to evaluate our multimodal group-based treatment program regarding a change of psychiatric burden, a change of protective factors, and possible differences in therapy outcome for patients with or without cPTSD.

**Methods:** The group-based treatment for patients with trauma-related disorders was examined in 66 patients who filled out the following questionnaires in the first and in the last week of treatment: Essen Trauma Inventory (ETI), Screening for complex PTSD (SkPTBS), Patient Health Questionnaire—somatization module (PHQ-15), Beck Depression Inventory—Revised (BDI-II), Posttraumatic Growth Inventory (PTGI), and Questionnaire on social support (F-SozU).

**Results:** The treatment was shown to significantly reduce depressive symptoms (p < 0.001, d = -0.536) and increase posttraumatic growth (New Possibilities: p = 0.004, d = 0.405; Personal Strength: p = 0.005, d = 0.414). For patients with cPTSD, depressive (p = 0.010, d = -0.63) as well as cPTSD symptoms (p = 0.020, d = -0.796) were significantly reduced; perceived social support was increased after day clinic treatment (p = 0.003, d = 0.61). Contrary to our expectations, somatoform symptoms were increased after therapy.

**Conclusions:** The present work expands previous research by demonstrating that multimodal group-based, day clinic treatment is effective in the treatment of trauma-related disorders, also in their complex form.

## Introduction

The prevalence of traumatic experiences throughout lifetime is high: in the US national comorbidity survey, 60.7% of men and 51.2% of women reported having experienced at least one traumatic event in their lifetime (1). Not all of these persons develop a psychiatric disease in the aftermath of trauma. However, 10% to 50% of survivors may develop a posttraumatic stress disorder (PTSD), depending on the type of the traumatic event (e.g., after automobile accidents or after rape, respectively) (2), or react with depressive, anxiety, or somatoform symptoms (2) or complex profiles. Other studies reported lower PTSD rates after various traumatic experiences (e.g., 19% after rape) (3). In a nationwide representative German sample, the 1-month prevalence rate for PTSD in the whole population (irrespective of having been confronted with a traumatic event) was 1.5% and for complex PTSD (cPTSD) 0.5% (4). For these persons, specialized treatment of trauma-related disorders is of utmost relevance.

The effectiveness of many psychotherapeutic treatments of PTSD and other trauma-related disorders is considered proven (5, 6, 7). Additionally, group-based programs have been examined and were shown to reduce trauma-related symptomatology (8, 9). For example, Sloan et al. (10) conducted a meta-analysis for group-based treatment programs and showed a small but significant effect. Treatment approaches combining individual and group therapy have rarely been examined, despite promising effects that were yielded by addressing more than one treatment target area, as Sloan et al. (11) stated. The authors concluded that more work is needed to examine the combination of group and individual treatment. In a meta-analysis with military samples, group-only therapy formats were shown to be less effective than individual-only or combinatory formats (12). Our study investigates a multimodal therapy program combining individual and group-based psychotherapy in a day clinic setting.

Previous studies supported evidence for the effectiveness of inpatient treatment programs for trauma-related disorders (13, 14). In Germany, the discipline of psychosomatic medicine and psychotherapy provides inpatient multimodal treatment, which is reimbursed by health insurance and offers a dense program of different therapy modules. Despite the wide use of such programs in German psychosomatics departments and clinics, only very few studies investigated their therapy outcomes. Lampe et al. (15, 16) showed a significant reduction of depressive symptoms as well as an increase in self-calming ability in inpatients with a history of sexual abuse in childhood treated over 6 weeks. No differences to the wait-list control group were found for PTSD, anxiety, and somatization symptoms. In a follow-up 2 years later, a significant reduction of overall symptom severity, PTSD, and depressive symptoms as well as an increase in self-calming ability were reported (16). Other examinations of inpatient treatment programs found significant improvements in dissociation, stress reaction and defense mechanisms (17), reductions of PTSD symptoms and improvements concerning feelings of self-esteem, freedom, and security (18) and improvements in PTSD, depressive and dissociative symptoms, as well as in interpersonal problems and general psychopathology (19). These improvements were found to be stable over a 1-year follow-up period. Lower effects were found for patients with complex dissociative disorders (19). In a following investigation, the authors showed dissociative symptoms and interpersonal problems previous to admission as well as their interaction to predict poorer therapy outcome (20).

To our knowledge, this is the first study to examine a multimodal, group-based day clinic treatment for trauma-related disorders. Day clinic programs have several advantages over inpatient treatment: Patients are still connected to their everyday life, which strengthens transfer of learned techniques and facilitates working on important problems or difficulties that would not be present during inpatient treatment. Patients can stay in their well-known environment and are able to spend evenings and nights in their own home, which may reflect an important safety anchor. Apart from that, day clinic programs are more cost-effective than inpatient treatments. Given these advantages, it is important to investigate the effectiveness of such treatment programs.

A growing number of studies pay attention to the question if therapy outcome differentiates for patients with classic versus complex PTSD and if treatment programs therefore need to account for this difference. cPTSD describes a symptom pattern that goes beyond classic PTSD symptoms and includes difficulties in emotion regulation (e.g., increased emotional reactivity, self-harming behavior), alterations in self-concept, e.g., disturbed feeling of identity, belief to live a shattered life or to be worthless, permanent feelings of guilt and shame, and relationship problems, e.g., inability to trust others or to be in a stable relationship (21, 22, 23). cPTSD is caused by severe interpersonal trauma that often lasted for a long period, such as sexual abuse in childhood or other forms of violence (21, 22, 24). To date, the two classification systems of ICD-11 and DSM-5 are not congruent in their definitions of PTSD or in their inclusion of the concept of cPTSD. cPTSD is not an official concept according to DSM-IV and DSM-5, but will be included in ICD-11. European countries differ in which classification system they use. Until now, the concept of cPTSD has been coded under different names in the diagnostic systems [e.g., Disorders of Extreme Stress Not Otherwise Specified (DESNOS)] and there is an ongoing debate about the significance and diagnostic entity of cPTSD [for an overview see (25)]. Although the symptom pattern of cPTSD has been examined for many years, there is still a controversy about presence, clinical significance, and treatment implications (26). It is unclear whether cPTSD patients are able to tolerate and benefit from first-line PTSD treatments; those often do not target all relevant psychopathology of cPTSD, e.g., affect regulation or interpersonal problems (24). Many studies excluded patients with complex clinical profiles (e.g., sexual abuse in childhood, personality disorders), so that only a limited amount of literature exists concerning the question whether cPTSD is a negative prognostic factor for therapy outcome (27).

A meta-analysis concerning treatments for child abuse-related cPTSD found a mean effect size of 1.7 (1.3 in the intention-to-treat-analysis) (24). cPTSD patients showed less treatment gain than non-complex PTSD patients as well as lower recovery rates and a lower improvement rate. Generally, only a minority of patients reached criteria for significant improvement after therapy. Another recent meta-analysis (28) indicates that group treatments for adults with symptoms associated with cPTSD are effective in reducing several psychopathological factors (e.g., PTSD symptoms, depression, and psychological distress). In an examination of an inpatient multimodal treatment program for patients with cPTSD, Kratzer et al. (29) found a reduction of PTSD, depressive, somatoform, and anxiety symptoms as well as an increase in well-being, self-efficacy, and mindfulness. They concluded that trauma-focused therapy is also applicable for patients with cPTSD. Cloitre et al. (26) recommended a phase-based approach for patients with cPTSD in their expert consensus statement for the International Society for Traumatic Stress Studies. A review of the existing literature showed effects ranging from g = -0.90 for cognitive behavioral therapy (CBT) to g = -1.26 for eye movement desensitization and reprocessing (EMDR) therapy with low number-needed-to-treat (NNT) indices (30). A moderator analysis showed childhood onset trauma to be associated with poorer outcome. The authors recommended exploring whether there are differences in outcome between patients fulfilling criteria for cPTSD and those who do not meet the diagnosis and to investigate the effectiveness of interventions using a dedicated measure for cPTSD, which is an aim of the present study.

Protective factors were also investigated in the present study. As Sloan et al. (11) stated, outcome measures other than PTSD, for example social functioning, should be investigated in clinical studies. Treatments for PTSD aim to enhance psychosocial functions observed in resilient individuals such as social support, emotion regulation, positive affect, meaning making, and post-traumatic growth. Whether this goal is actually achieved has rarely been investigated; only a few studies included resilience factors as outcome variables (31).

An advantage of group-based treatment is the enhancement of social support and contact to other patients with a comparable history (e.g., cohesion, universality) (11, 32), but perceived social support has rarely been examined as outcome variable. Perceived social support by significant others was shown to moderate treatment outcome, indicating that higher perceived social support at the beginning is associated with greater reduction of PTSD symptoms in the treatment group (33). The authors questioned if perceived social support can be increased by therapy and stated that more research is needed in this field. In another examination, a positive association was found between social support during therapy and reduction of PTSD symptoms, as well as an increase in social support (34).

Another important protective factor is posttraumatic growth (PTG). The term describes “positive psychological change experienced as a result of the struggle with highly challenging life circumstances” (35) and consists of five factors: personal strength, new possibilities, relating to others, appreciation of life, and spiritual change (36). PTG and negative consequences of trauma such as PTSD are separate dimensions whose association is not definitely clear: Some studies found a positive (37, 38), others a negative association (39, 40), a third group detected no correlation at all (41, 42). Some authors assumed a curvilinear relationship in form of an inverted “U,” a Janus Faced improvement, so that minimal or extreme PTSD symptoms relate to a low extent of PTG (40). Previous research was able to show an increase of PTG or different subscales after treatment (37, 39, 40).

The aim of the present study was to evaluate our multimodal group-based treatment program regarding a change of psychiatric burden, a change of protective factors, and possible differences in therapy outcome for patients with or without cPTSD.

We hypothesized a reduction of psychiatric burden measured by PTSD, cPTSD, depressive and somatoform symptoms, as well as an increase in perceived social support and PTG. Further, we explored the following question: Are there differences concerning therapy outcomes between patients with cPTSD versus patients with non-complex trauma-related disorders?

## Materials and Methods

### Treatment Description

Since 2014, the day clinic of the Department of Psychosomatic Medicine and Psychotherapy at the University Hospital Erlangen offers a specialized treatment of trauma-related disorders. Trauma-related disorders include PTSD in its classic or complex form as well as anxiety, affective, somatoform, or personality disorders that relate to traumatic experiences in the past. Exclusion criteria are acute psychosis, acute suicidality, present substance abuse or dependence, clear underweight (BMI < 17 kg/m^2^), unstable social conditions such as homelessness, a journey to the day clinic of more than 1 h, contact to the offender, or not being able to participate in groups (e.g., extreme dissociation).

Trauma-focused therapy is carried out in a closed group format, so that seven patients enter and complete treatment together. Program modules are the following: psychotherapy in individual (1 × 50 min) and group (2 × 100 min) format, trauma-specific psychoeducation (1 × 100 min), skills training (2 × 60 min), mindfulness and relaxation methods (2 × 50 min), art therapy (1 ×  120 min), concentrative movement therapy (CMT, 1 × 120 min), and pharmacological therapy if needed. Treatment quality is ensured by weekly team meetings and internal as well as external supervision. Psychotherapeutic treatment includes cognitive behavioral as well as psychodynamic techniques. Stabilizing and confrontational methods are used, the amount of each is chosen by the therapists on the basis of therapy goals and the patient`s status in the therapeutic process. Stabilization includes not only psychoeducation and training in affect regulation but also employment of problem- and solution-oriented coping techniques, distancing methods, and cognitive restructuring of trauma-related thoughts. Symptoms that lower psychosocial functioning (e.g., interactional problems) are treated.

### Design and Procedure

Data were assessed in a longitudinal, naturalistic design. Every patient who started therapy in the group for patients with trauma-related disorders was asked to participate in our study. Written informed consent was obtained from every participant. Seventy-three patients were asked to participate; 66 (90.4%) gave their written consent. The study was approved by the ethics committee of the Medical Faculty of the Friedrich-Alexander University Erlangen-Nürnberg (FAU) (153_18B). After being informed about study course, goals, and potential risks (e.g., being reminded of traumatic experiences while filling out questionnaires), participants completed the questionnaires described in section *Instruments* (T1 in week 1). These were also used for therapy planning and diagnosing by the psychotherapists that carried out therapy. In week 8, participants completed the same questionnaires again (T2).

### Instruments

The Essen Trauma Inventory (ETI) (43) contains a list of potentially traumatic experiences, questions concerning objective and subjective threat to life (criteria A1 and A2), and questions about symptoms on the subscales intrusion, hyperarousal, avoidance, and dissociation. Symptoms are rated on a 4-point Likert-type scale ranging from “0 = never” to “3 = very often”. Clinically relevant PTSD is indicated by existence of a traumatic experience, a fulfilled A1 and A2 criteria as well as a symptom sum score ≥ 27. As the ETI is based on DSM-IV, the cut-off only covers the sum of the three subscales intrusion, hyperarousal, and avoidance; the items concerning the dissociation subscale are not involved in the cut-off score. The ETI was proven to be a reliable and valid questionnaire (43). In our study, Cronbach's alpha was α = 0.943 for T1 and α = 0.953 for T2.

The Screening for complex PTSD (in German, SkPTBS) (44) is a questionnaire that assesses potentially traumatic experiences, risk and protective factors like age at onset, frequency and duration, type of causation (e.g., family member, accident) and complex PTSD symptoms. These include difficulties in affect and impulse control (e.g., self-calming ability, anger control), interactional problems (e.g., ability to trust another person), negative self-image (e.g., feelings of guilt, belief to live a shattered life), and dissociative symptoms. Symptoms are rated on a 7-point Likert scale ranging from “0 = does not apply at all” to “6 = totally fits”. A high total value therefore reflects a high amount of complex PTSD symptomatology. Comparative values and a division into very high, high, and low risk for complex PTSD are also given. Dorr et al. (44) showed the scale to be reliable, one-dimensional, and valid. Cronbach's alpha was α = 0.891 for T1 and α = 0.904 for T2.

The Patient Health Questionnaire: somatization module (PHQ-15) (45) consists of 13 items measuring somatic symptoms with the response options “not bothered at all” (0), “bothered a little” (1), or “bothered a lot” (2). Two additional items from the depression module are included in the sum score; these assess sleeping disorders and tiredness. The possible range of the resulting sum score is 0 to 30 points. Sum scores of 5, 10, and 15 represent cut-offs for mild, moderate, and severe levels of somatization. For the PHQ-15, good psychometric properties have been demonstrated (46). In our study, Cronbach's alpha was α = 0.772 for T1 and α = 0.826 for T2.

The Beck Depression Inventory-Revised [BDI-II (47), German version (48)] is a 21-item questionnaire considering a variety of depressive symptoms such as sadness, feelings of guilt, insufficiency or worthlessness, and reduced interest in others as experienced within the last 2 weeks. Responses are rated on a 4-point scale ranging from 0 to 3 anchored with example sentences. Sum scores can be assigned to severity of depression symptoms using the following scheme: 0–8 no depression, 9–13 minimal, 14–19 mild, 20–28 moderate, and 29–63 severe depression. The authors demonstrated good reliability and validity for the BDI-II. Internal consistency in our study was α = 0.904 for T1 and α = 0.927 for T2.

The Posttraumatic Growth Inventory [PTGI (49), German version (50)] is a 21-item questionnaire that asks about positive change in the aftermath of a traumatic experience and consists of five subscales: New Possibilities (e.g., “I developed new interests.”), Relating to Others (e.g., “Having compassion for others.”), Personal Strength (e.g., “I discovered that I’m stronger than I thought I was.”), Appreciation of Life (e.g., “I changed my priorities about what is important in life.”), and Spiritual Change (e.g., “I have a stronger religious faith.”). Responses are rated on a 6-point Likert-type scale ranging from “0 = not at all” to “5 = very much”. Scores are calculated by adding the respective items with higher scores indicating higher PTG. The authors were able to show good psychometric properties for the PTGI and the corresponding German version. We adapted the instruction according to our study population: In an introductory sentence, we explained why patients are asked about positive changes after traumatic experiences to prevent feelings of not being understood or taken seriously in their sorrow. Patients were asked to name the most distressing traumatic event and their age at time of the experience. Internal consistency was α = 0.920 for T1 and α = 0.917 for T2.

The Questionnaire on social support (in German: Fragebogen zur sozialen Unterstützung, F-SozU) (51) is a self-rating instrument measuring perceived social support. We used the short version K-14 consisting of 14 items such as “There are people who share joy and sorrow with me” or “I have no problems finding someone who looks after my flat when I’m not there”. Answers to these statements are rated on a 5-point scale ranging from “0 = does not apply at all” to “4 = totally fits”. The authors recommend only calculating a total score due to small item numbers when calculating subscales. A factor analysis confirmed that all items load on one factor. The F-SozU K-14 demonstrated good reliability and validity. Cronbach’s alpha in our study was α = 0.952 for T1 and α = 0.956 for T2.

### Statistical Analysis

Statistical analysis was performed using SPSS 25 (IBM Statistics, Chicago, IL, USA). After analyzing missing values with Little’s MCAR test (52), values that were missing completely at random were replaced using expectation maximization (EM) method (53). Dropout was very low (N = 2, 3.0%), so that missing T2 scores could be replaced using the “last value carried forward” method (11). Hence, results are based on an intention to treat analysis (54, 55). An analysis of outlier scores indicated that the data contained only few outliers. These were left in the sample because of the plausibility of the scores and the natural variability in the sample.

Division of patients with complex trauma-related disorder versus non-complex disorders was made using the SkPTBS scores (see instruments). As n = 49 patients (75.4%) were classified as being on a high risk of having complex PTSD, we decided to use the more strict cut-off of being on a very high risk for complex PTSD (e.g., reaching a score of 28,19 or higher).

To profile the sociodemographic characteristics of the total sample and the subsamples, the following descriptive statistics were computed: means, standard deviations, ranges, and frequencies.

Evaluation of our treatment program is provided for the total sample as well as for the sub-groups of patients with or without complex PTSD, respectively. For the total sample, comparisons were analyzed using t-tests. For the sub-group analyses, we first verified if data was normally distributed because of low sample sizes. If values were not normally distributed, we conducted non-parametric Wilcoxon tests instead of t-tests. Significance level in all analyses was p ≤ 0.05. To measure the effect size, we computed Cohen's d.

## Results

### Sample Characteristics

Our study included N = 66 patients: 55 females (83.3%) and 11 males (16.7%). Males were significantly older (M = 50.00, SD = 9.26) than females (M = 38.07, SD = 13.18; t = -3.60, df = 19.15, p = 0.002). Treatment diagnoses given on the basis of clinical impression and questionnaires are depicted in **Table 1**. Of the 53 patients diagnosed with a PTSD, 22 were diagnosed with the appendix “complex.” The diagnosis of emotionally unstable personality disorder was given in five of the seven patients with the main diagnosis personality disorder. In a further patient, this was a comorbid diagnosis. Comorbidity with other psychiatric disorders as well as the number of diagnoses were high (see **Table 1**). Information on pharmacological treatment was gathered from patient records. Forty-two patients (63.6%) were treated with antidepressants, 18 (27.3%) with neuroleptic medication, 5 (7.6%) received benzodiazepines, 24 (36.4%) took analgesics, and 48 (72.2%) received various pharmacological treatment for chronical conditions.

**Table 1:** Distribution of main diagnoses, comorbidity, and number of diagnoses.

	N	%
**Main Diagnosis**		
Posttraumatic stress disorder	46	69.70
Other reactions to severe stress	7	10.61
Personality disorders	7	10.61
Depressive disorders	5	7.58
Obsessive compulsive disorder	1	1.52
**Comorbid Diagnoses**		
Depressive disorders	60	90.91
Somatoform disorders	15	22.73
Anxiety disorders	11	16.67
Posttraumatic stress disorder	7	10.61
Obsessive compulsive disorder	5	7.58
Personality disorders	4	6.06
Eating disorders	4	6.06
Substance abuse	3	4.55
Dissociative disorders	2	3.03
Impulse control disorders	1	1.52
Hyperkinetic disorders	1	1.52
**Number of diagnoses^1^**		
1 diagnosis	1	1.52
2 diagnoses	31	46.97
3 diagnoses	19	28.79
4 diagnoses	11	16.67
5 diagnoses	4	6.06

^1^Without consideration of possible somatic diagnoses.

Sociodemographic variables and data concerning pretreatment are shown in **Table 2**. No significant differences between the subgroups were found, except for the frequency of pretreatment inpatient admissions: There was a significant association between being on a very high risk for cPTSD and reporting inpatient treatment in the past [χ² (1) = 4.196, Fisher's exact test p = 0.048]. cPTSD patients more often reported inpatient treatment before being admitted to our group treatment.

**Table 2:** Sociodemographic and pretreatment variables.

	Total sample (N = 66)		Females (N=55)		Males (N = 11)		Complex PTSD (N=28)		Non-Complex (N = 37)	
	M (SD)	Range	M (SD)	Range	M (SD)	Range	M (SD)	Range	M (SD)	Range
Age	40.06 (13.32)	18-61	38.07 (13.18)	18-61	50.00 (9.26)	27-61	40.14 (13.25)	18-61	40.05 (13.74)	18-61
	N	%	N	%	N	%	N	%	N	%
**Marital status**										
Single	26	41.9	23	45.1	3	27.3	11	44.0	15	41.7
Unmarried, living together	4	6.5	4	7.8	0	0.0	1	4.0	3	8.3
Married, living together	21	33.9	14	27.5	7	63.6	7	28.0	13	36.1
Married, but separated	3	4.8	3	5.9	0	0.0	2	8.0	1	2.8
Divorced	7	11.3	6	11.8	1	9.1	3	12.0	4	11.1
Widowed	1	1.6	1	2.0	0	0.0	1	4.0	0	0.0
**Current Relationship**										
Yes	41	66.1	34	65.4	7	70.0	16	59.3	24	70.6
No	21	33.9	18	34.6	3	30.0	11	40.7	10	29.4
**Education**										
No school-leaving qualification	3	4.8	3	5.9	0	0.0	2	8.0	1	2.7
Qualification after 9 years	16	25.8	12	23.5	4	36.4	8	32.0	8	22.2
Qualification after 10 years	22	35.5	19	37.3	3	27.3	9	36.0	13	36.1
Qualification after 13 years (University entrance diploma)	20	32.3	16	31.4	4	36.4	6	24.0	14	38.9
Pretreatment*										
Outpatient psycho-therapeutic	39	59.1	30	54.5	9	81.8	19	67.9	20	54.1
Outpatient psychiatric	20	30.3	15	27.3	5	45.5	10	35.7	10	27.0
Inpatient treatment	30	45.5	26	47.3	4	36.4	17	60.7	13	35.1
Physicians with other specializations	11	16.7	34	65.4	3	27.3	5	17.9	6	16.2

Varying sample sizes; *multiple references possible.

### Traumatic Events

Sixty-five patients (98.5%) reported having experienced and/or witnessed traumatic events (TE). The one person who reported not having experienced any TE was diagnosed with a complex PTSD, so that self-report on the ETI and reported biographical information in psychotherapy did not match and the person was left in the sample for the following analyses. **Table 3** shows the distribution of the most frequent TE for the total sample and the different subgroups as well as the amount of patients having experienced persistent TE and sexual violence in their infancy.

**Table 3:** Experiences of traumatic events (TE).

	Total sample (N = 66)	Women (N = 55)	Men (N = 11)	Non-complex subgroup (n = 37)	Complex PTSD subgroup (n = 28)
Most frequent traumatic events (TE)^1^	-Serious illness (N = 46, 69.7%)	-Neglect (N = 39, 70.9%)	-Serious illness (N = 8, 72.7%)	-Serious illness (N = 28, 75.7%)	-Neglect (N = 22, 78.6%)
	-Neglect (N = 43, 65.2%)	-Serious illness (N = 38, 69.1%)	-Natural catastrophe (N = 7, 63.6%)	-Physical violence by family member (N = 22, 59.5%)	-Serious illness (N = 18, 64.3%)
	-Physical violence by family member (N = 41, 62.1%)	-Physical violence by family member (N = 34, 61.8%)	-Physical violence by strangers (N = 7, 63.6%)	-Neglect (N = 20, 54.1%)	-Physical violence by family member (N = 18, 64.3%)
			-Physical violence by family member (N = 7, 63.6%)		
Having experienced persistent TE^2^	N = 45 (75.0%)	N = 38 (69.1%)	N = 7 (63.6%)	N = 26 (70.3%)	N = 19 (67.9%)
Having experienced sexual violence in their infancy	N = 41 (62.1%)	N = 39 (70.9%)	N = 2 (18.2%)	N = 19 (51.4%)	N = 21 (75.0%)

^1^as reported in the ETI, ^2^e.g many years as reported in the SKPTBS.

### Treatment Evaluation

As shown in **Table 4**, depressive symptoms were significantly reduced at T2 (t = 3.977, df = 64, p < 0.001) with an effect size of Cohen’s d = -0.536, indicating a medium effect. Also, two dimensions of the PTGI significantly increased (see **Table 5**): New Possibilities (t = -2.993, df = 49, p = 0.004, Cohen’s d = 0.405) and Personal Strength (t = -2.919, df = 50, p = 0.005, Cohen’s d = 0.414). The PTGI total score was found to show a trend to be increased (t = -1.993, df = 49, p = 0.052, Cohen’s d = 0.277), as too did perceived social support (t = -1.784, df = 65, p = 0.079, Cohen’s d = 0.216). Contrary to our hypothesis, we found a significant increase in the somatization scores (see **Table 4**). No other indices showed a significant change over time.

**Table 4:** Treatment evaluation for the total sample: symptom scores.

Variable	T1	T2	Comparison		
	M (SD)	M (SD)	t	p	d
ETI total	39.45 (16.65)	39.58 (17.88)	-0.114	0.910	0.014
ETI PTSD	31.91 (12.31)	31.79 (12.91)	0.125	0.901	-0.017
ETI intrusion	9.52 (4.01)	9.85 (4.42)	-0.917	0.363	0.120
ETI avoidance	12.35 (5.40)	12.23 (5.57)	0.280	0.780	-0.035
ETI hyperarousal	10.04 (3.96)	9.71 (3.72)	0.933	0.355	-0.114
ETI dissociation	7.54 (5.23)	7.79 (5.64)	-0.658	0.513	0.035
SkPTBS	35.18 (33.21)	35.38 (33.72)	-0.063	0.950	0.008
BDI-II	30.43 (11.65)	26.21 (13.27)	3.977	**<0.001**	-**0.536**
PHQ somatization	13.70 (5.64)	15.28 (6.17)	-3.418	**0.001**	**0.457**
PHQ pain	6.00 (3.13)	6.67 (3.23)	-2.623	**0.011**	**0.333**
PHQ other somatization	4.28 (2.87)	5.41 (3.01)	-3.752	**<0.001**	**0.485**

ETI, Essen Trauma Inventory; ETI total, sum score of all four subscales; ETI PTSD, sum score calculated on the basis of the three subscales that are relevant for the cut-off for a PTSD diagnosis; SkPTBS, Screening for complex PTSD; BDI-II, Beck Depression Inventory-Revision; PHQ, Patient Health Questionnaire; PHQ pain, items asking about experiencing pain; PHQ other somatization: items asking about other somatoform symptoms; varying sample sizes and degrees of freedom (df).

Bolded texts highlight significant differences and effect sizes.

**Table 5:** Treatment evaluation for the total sample: protective factors.

Variable	T1	T2	Comparison		
	M (SD)	M (SD)	t	p	d
PTGI total	40.54 (17.49)	45.22 (16.90)	-1.993	0.052	0.277
PTGI Relating to Others	15.65 (6.81)	16.92 (6.96)	-1.336	0.188	0.194
PTGI New Possibilities	8.80 (5.23)	10.86 (4.73)	-2.993	**0.004**	**0.405**
PTGI Personal Strength	7.35 (4.54)	9.20 (4.62)	-2.919	**0.005**	**0.414**
PTGI Spiritual Change	1.96 (2.80)	2.14 (2.86)	-0.669	0.507	0.096
PTGI Appreciation of Life	6.75 (3.55)	6.88 (3.50)	-0.285	0.777	0.038
F-SozU	33.53 (14.40)	35.22 (13.95)	-1.784	0.079	0.216

PTGI, Posttraumatic Growth Inventory; F-SozU, Questionnaire on social support; varying sample sizes and degrees of freedom (df).

Bolded texts highlight significant differences and effect sizes.

Patients with and without cPTSD differed significantly on T1 und T2 symptom scores: cPTSD patients had significantly higher scores on almost all symptom scores (e.g., ETI, BDI-II). cPTSD patients also had significantly lower scores at T1 for perceived social support. Data are shown in corresponding tables in the **Supplementary Material**.

We then conducted a sub-group analysis and evaluated the treatment program for patients with complex PTSD versus those with non-complex trauma-related disorders, respectively. For results, see **Tables 6**, **7**, **8**, and **9**.

**Table 6:** Treatment evaluation for the non-complex sub-group (N = 37): symptom scores.

Variables	Non-complex trauma-related disorder				
	T1	T2	Comparison		
	M (SD)	M (SD)	t	p	d
ETI total	33.04 (15.47)	31.52 (17.78)	1.019	0.315	-0.188
ETI PTSD	27.34 (11.64)	26.03 (13.32)	1.142	0.261	-0.208
ETI intrusion	8.42 (3.85)	8.28 (4.76)	0.331	0.743	-0.063
ETI avoidance	9.93 (4.79)	9.35 (5.24)		0.455	-0.184
ETI hyperarousal	8.99 (3.94)	8.40 (4.05)	1.351	0.185	-0.227
ETI dissociation	5.69 (4.95)	5.49 (5.30)		0.700	-0.066
SkPTBS	9.40 (8.32)	19.72 (24.29)		**0.022**	**1.169**
BDI-II	25.65 (10.52)	21.77 (12.49)	2.989	**0.005**	-**0.554**
PHQ somatization	12.50 (6.09)	13.52 (6.84)	-1.629	0.112	0.289
PHQ pain	5.57 (3.46)	6.00 (3.47)	-1.341	0.189	0.226
PHQ other somatization	3.63 (3.01)	4.37 (3.18)		0.078	0.314

ETI, Essen Trauma Inventory; ETI total, sum score of all four subscales; ETI PTSD, sum score calculated on the basis of the three subscales that are relevant for the cut-off for a PTSD diagnosis; SkPTBS, Screening for complex PTSD; BDI-II, Beck Depression Inventory-Revision; PHQ, Patient Health Questionnaire; PHQ pain, items asking about experiencing pain; PHQ other somatization, items asking about other somatoform symptoms; varying sample sizes and degrees of freedom (df), Wilcoxon test where no t is defined.

Bolded texts highlight significant differences and effect sizes.

**Table 7:** Treatment evaluation for the non-complex sub-group (N = 37): protective factors.

Variables	Non-complex trauma-related disorder				
	T1	T2	Comparison		
	M (SD)	M (SD)	t	p	d
PTGI total	40.63 (16.37)	45.97 (15.61)	-1.857	0.074	0.332
PTGI Relating to Others	15.86 (5.99)	17.69 (6.94)	-1.549	0.133	0.313
PTGI New Possibilities	8.07 (4.89)	10.70 (4.50)	-3.117	**0.004**	**0.547**
PTGI Personal Strength	7.78 (4.79)	10.03 (4.64)	-2.501	**0.018**	**0.443**
PTGI Spiritual Change	1.74 (2.49)	1.77 (2.63)		0.975	0.018
PTGI Appreciation of Life	7.13 (3.43)	7.06 (3.45)	0.123	0.903	-0.024
F-SozU	38.21 (13.12)	37.50 (13.85)		0.813	-0.107

PTGI, Posttraumatic Growth Inventory; F-SozU, Questionnaire on social support; varying sample sizes and degrees of freedom (df), Wilcoxon test where no t is defined.

Bolded texts highlight significant differences and effect sizes.

**Table 8:** Treatment evaluation for the complex PTSD sub-group (N = 28): symptom scores.

Variables	Very high risk for Complex PTSD				
	T1	T2	Comparison		
	M (SD)	M (SD)	t	p	d
ETI total	47.92 (14.71)	50.15 (12.02)		0.361	0.213
ETI PTSD	37.88 (10.88)	39.34 (7.83)		0.572	0.170
ETI intrusion	10.85 (3.89)	11.95 (2.98)		0.066	0.330
ETI avoidance	15.47 (4.65)	16.00 (3.46)		0.694	0.133
ETI hyperarousal	11.56 (3.51)	11.39 (2.51)		0.674	-0.053
ETI dissociation	10.04 (4.64)	10.81 (4.74)		0.199	0.269
SkPTBS	69.24 (20.19)	56.07 (33.62)		**0.020**	-**0.796**
BDI-II	37.51 (9.40)	32.49 (12.15)	2.759	**0.010**	-**0.630**
PHQ somatization	15.71 (4.32)	18.09 (3.76)		**0.002**	**0.662**
PHQ pain	6.65 (2.61)	7.75 (2.57)	-2.493	**0.020**	**0.489**
PHQ other somatization	5.23 (2.48)	6.95 (2.02)	-3.819	**0.001**	**0.690**

ETI, Essen Trauma Inventory; ETI total, sum score of all four subscales; ETI PTSD, sum score calculated on the basis of the three subscales that are relevant for the cut-off for a PTSD diagnosis SkPTBS, Screening for complex PTSD; BDI-II, Beck Depression Inventory-Revision; PHQ, Patient Health Questionnaire; PHQ pain, items asking about experiencing pain; PHQ other somatization, items asking about other somatoform symptoms; varying sample sizes and degrees of freedom (df), Wilcoxon test where no t is defined.

Bolded texts highlight significant differences and effect sizes.

**Table 9:** Treatment evaluation for the complex PTSD sub-group (N = 28): protective factors.

Variables	Very high risk for Complex PTSD				
	T1	T2	Comparison		
	M (SD)	M (SD)	t	p	d
PTGI total	40.40 (19.50)	44.10 (19.04)	-0.909	0.375	0.201
PTGI Relating to Others	15.35 (8.00)	15.80 (7.01)	-0.284	0.780	0.060
PTGI New Possibilities	9.90 (5.65)	11.10 (5.16)	-1.031	0.316	0.221
PTGI Personal Strength	6.70 (4.14)	7.90 (4.39)	-1.494	0.152	0.345
PTGI Spiritual Change	2.30 (3.26)	2.70 (3.16)		0.406	0.183
PTGI Appreciation of Life	6.15 (3.75)	6.60 (3.63)	-0.482	0.635	0.106
F-SozU	27.15 (14.02)	32.00 (13.90)	-3.243	**0.003**	**0.610**

PTGI, Posttraumatic Growth Inventory; F-SozU, Questionnaire on social support; varying sample sizes and degrees of freedom (df), Wilcoxon test where no t is defined.

Bolded texts highlight significant differences and effect sizes.

For patients with non-complex trauma-related disorders, we found a significant reduction of depressive symptoms (t = 2.989, df = 36, p = 0.005, d = -0.554, see **Table 6**) as well as a significant increase on two PTGI scales (see **Table 7**): New Possibilities (t = -3.117, df = 29, p = 0.004, d = 0.547) and Personal Strength (t = -2.501, df = 30, p = 0.018, d = 0.443). The PTGI total score showed an increase (t = -1.857, df = 29, p = 0.074, d = 0.332). On the other hand, somatoform symptoms tended to be increased (p = 0.078, d = 0.314), but this increase was not statistically significant. Also complex PTSD symptoms showed a significant increase (p = 0.022, d = 1.169).

For patients with complex PTSD, results showed quite a different pattern: Depressive symptoms (t = 2.759, df = 26, p = 0.010, d = -0.63) as well as complex PTSD symptoms (p = 0.020, d = -0.796) were significantly reduced (see **Table 8**). Also, perceived social support showed a highly significant increase (t = -3.243, df = 27, p = 0.003, d = 0.61, see **Table 9**). All three somatization indices showed a significant increase in somatoform symptoms (see **Table 8**).

## Discussion

### Main Results

The major outcome of this survey is that our multimodal, group-based therapy program for patients with trauma-related disorders is associated with reductions of depressive symptoms and increases in PTG. Contrary to our expectations, somatoform symptoms increased across time. After dividing the sample into patients with and without complex PTSD, important differences in therapy outcome were observed: For patients with non-complex trauma-related disorders, we found a reduction of depressive symptoms and an increase in PTG. Somatoform symptoms increased in a trend. For patients with cPTSD, we found a reduction of depressive symptoms as well as of cPTSD symptoms. Perceived social support was significantly increased in this group. Somatoform symptoms increased over time.

### Positive Changes After Therapy

The observed reduction of depressive symptoms (and cPTSD symptoms for those patients with cPTSD) and increase in protective factors like PTG and perceived social support align with other studies describing positive treatment effects after different group-based therapy programs (14, 17, 18). Similar to our study, Lampe and colleagues (15) reported a reduction of depressive symptoms; PTSD scores did not differ significantly from those in the wait list group after their 6 week therapy program. Significant group differences in PTSD and somatoform symptoms were only found in a follow-up study after 2 years (16).

When interpreting our results, one can see that body-related factors such as somatoform symptoms were not reduced, whereas factors referring to relationships and interpersonal skills (e.g., perceived social support, cPTSD symptoms, New Possibilities and Personal Strength) were strengthened after the group-based therapy program: those factors that were addressed directly by the therapy interventions showed a significant positive effect. The aforementioned changes after therapy strengthen patients' level of functioning, self-efficacy, belief in their own abilities, courage to face life and form a more positive perspective to the future (as indicated by the items of BDI-II, SkPTBS and PTGI, e.g. hopelessness, feelings of being shattered, perception of new possibilities, feelings of personal strength). These changes can be assumed to increase patients' quality of life. The achieved improvements prepare patients to face their PTSD symptoms in everyday life. The reduction of depressive symptoms may be related to the increase in PTG: hopelessness and lack of perspective are replaced by perceptions of new possibilities, low self-esteem and feelings of weakness are replaced by personal strength. As Shalev (56) stated, the reduction of depressive symptoms is central to patients' well-being, and also a partial therapy outcome should be seen as a breakthrough for chronically traumatized patients. The author argued that reducing PTSD symptoms should not be used as main criterion for the success of an intervention and that the addressing of dysfunctional affect and social competence is important. These are two of the main factors of our treatment program.

### Changes in PTSD and cPTSD

To explain the finding that the manifestation of posttraumatic symptoms measured by ETI did not show significant improvements in the sample, it is also important to keep in mind that our study population consists of severely traumatized patients: 75.0% of the participants reported having experienced the TE for a long period, 59.1% reported having experienced sexual violence in their infancy. Sloan et al. (10) found in their meta-analysis that studies with a high percentage of patients with a history of child sexual abuse show smaller effect sizes than studies with participants reporting other traumatization. This effect is strengthened by a recent meta-analysis conducted by Karatzias et al. (30). The authors found childhood onset trauma to be associated with poorer outcome. Most of our participants reported having experienced more than one TE. Additionally, although contact to the offender was an exclusion criterion, many patients still had contact to people connected with the TE because traumatization often took place within the family. Hence, although patients had no contact to the offender, they still met people who knew about the traumatic events, which may be a trigger for patients. It can be concluded that these patients remain under the influence of the family atmosphere in which they were humiliated. However, increased social support was associated with decreased symptom load. We conclude that patients who could mobilize support could benefit from this and stabilize mentally.

These factors may have influenced therapy and treatment success negatively. Therefore, the achieved stabilization of our patients reflects an important improvement of their well-being and a basis for following outpatient treatment. As Stalker et al. (57) stated, a short term therapy program may not be sufficient to achieve clinically significant change because of the potential chronicity of PTSD, so that rather a partial than a full success could be reached [also see (32)]. Nonetheless, a short term program can encourage patients to join in additional forms of psychotherapy (58). An interval treatment, as also described by Sachsse et al. (17), is optional for patients treated in the previously described group and is adopted by quite a few.

The finding that cPTSD symptoms were significantly reduced at the end of therapy for cPTSD patients reflects the difference between classic and complex PTSD symptoms: whereas classic PTSD symptoms were not significantly changed, complex PTSD symptoms, which include difficulties in interactions with other people (see introduction), were reduced. We assume that in our group therapy program, patients were able to increase their social competencies and create positive experiences when interacting with other people. However, the ETI only measures classic PTSD symptoms, whose treatment possibly needs a higher amount of confrontational methods (trauma exposition, EMDR) as used in this therapy program, which could explain the lack of improvement on the ETI scores.

It must be considered that our concept is aimed at stabilization first and confrontational methods are secondary. In Cloitre and colleagues' (59) STAIR program, PTSD symptoms were only reduced after the confrontation phase, not after stabilization and skills training. In contrast, several groups (8, 60, 61) reported no differences between confronting and stabilizing therapy programs. In the current model, therapists decide individually whether confrontation may be beneficiary and if it is to be conducted. In a current debate about the significance of stabilization in the treatment of trauma-related disorders, de Jongh et al. (62) presented a critical view on this concept. The authors reviewed the existing literature and argued that the research on stabilization is based on methodologically limited studies and that there is no clear evidence for a stabilization phase prior to the use of confrontational methods. Concerning this debate, one has to keep in mind that the term “stabilization” is not clearly defined: It is likely that stabilization describes different methods, procedures and therapy goals in different articles (63). In our treatment, stabilization included psychoeducation about the disorder, stress management and distancing methods. We further employed problem- and solution-oriented coping techniques and cognitive restructuring of trauma-related thoughts. Furthermore, symptoms of avoidance were discussed and symptoms that lower psychosocial functioning (e.g., interactional problems) were treated. Hence, “stabilization” not only included training in affect regulation and self-calming abilities but also focused on trauma-related contents and clearing of associations between traumatic experiences and present symptomatology or difficulties in everyday life. The present-centered therapy approach introduced by Schnurr et al. (60) includes similar contents and was shown to be an efficacious and safe treatment for trauma-related disorders (64, 65). Skipping the presented aspects and starting directly with confrontational methods, e.g. exposition, would decrease patients' well-being and increase the risk of additional problems (e.g., losing their job or deterioration of their relationship) (66). However, other studies also demonstrated that exposure treatment can be delivered to patients following multiple trauma and multiple treatment attempts (67).

### Increase of Somatoform Symptoms

The increase of somatoform symptoms may represent adverse effects of the intensive occupation with traumatic experiences. As Johnson and colleagues (68) reported, traumatic experiences may have been brought up, attention may have been directed to symptoms and patients may have been sensitized to observe potential symptoms more intensely. Additionally, the increase in somatoform symptoms in the course of therapy may be due to the fact that the patients came into the therapy with extensive neglect of medical problems and physical care, e.g. contacting doctors was worked out with some of them as a therapy goal. This circumstance may have caused the patients' attention on the body. Nevertheless, patients reported that talking about symptoms, etiology and relating factors was important to understand their disorder and symptoms. Many authors (30, 63, 69) highlight that it is necessary to also report adverse effects or deterioration after therapy. Another reason for the increase in somatoform symptoms and stagnation in PTSD symptoms is that our patients reported a very high extent of somatoform symptoms (M = 13.70) compared to the findings from Gräfe and colleagues (70; M = 6.4, 9.8, 9.7 for patients without psychiatric disorder, with psychiatric comorbidity and those in psychosomatic treatment, respectively). In addition, T2 assessment was conducted in the last week of treatment (week 8), in which patients had to disengage from the group and the therapists. As many patients told the therapeutic team, they felt themselves accepted often for the first time. Also, patients have to focus again on their everyday life with all requests and problems. Hence, it can be assumed that the end of this intensive therapy is a critical phase in patients' therapeutic process. These difficulties might have compromised the symptom ratings.

The aforementioned sensitization of patients to observe potential symptoms more intensely (68) may also explain the increase in cPTSD symptoms for patients with non-complex trauma-related disorders. It is likely that patients learned about their disorder through psychoeducation and clearing of associations between traumatic experiences and present symptomatology, so that they developed a higher understanding of their symptoms and were able to report them more explicitly at T2. Additionally, although the SkPTBS was examined to be a highly valid and usable instrument (44), it was only used in very few clinical studies until now. The increase of the scores for patients without cPTSD could be associated with further variables, which have not been examined in the present study. Future studies should explore the mechanisms underlying this finding.

### The Concept of cPTSD

As presented in the introduction, there is an ongoing debate about the concept of cPTSD (25). The results of this study highlight the differences between classic and complex PTSD. cPTSD can be seen as PTSD plus additional characteristics, which leads to a differential influence on therapeutic processes. The present results demonstrate that it is important to screen for and to differentiate between the two diagnoses as patients react differently in therapy. Without considering the concept of cPTSD, important differences in therapy outcome could be overlooked.

### Limitations and Strengths

Our study has some limitations that need to be considered. Due to organizational reasons it was not possible to include a control group in our study; hence, clear implications of causality cannot be drawn. Furthermore, symptom ratings are not based on structured interviews but on self-rating instruments.

Despite these limitations, our study has various strengths: It is one of the first to examine a multimodal, group-based day clinic treatment for patients with trauma-related disorders. Most previous studies examined special programs and focused on assessing symptomatology without paying attention to resilience factors. As such, our study expands previous research and has implications for therapy. The study provides high external validity and generalizability, reflects clinical practice and demonstrates how treatments work in field (17, 71). Also, the sub-group analysis concerning a division of patients in those with and without complex PTSD, respectively, is a strength of our study: In the current literature there is no consensus whether therapy programs for PTSD also function for patients with complex PTSD. As such, the results of our study further develop psychotherapy for patients with cPTSD and provide advice to adapt therapeutic work. Our study is also one of the first to examine a day clinic treatment program, which has various advantages for patients such as being connected to their everyday life or being able to spend evenings and nights at home, which may strengthen feelings of safety. Furthermore, day clinic programs are more cost effective than inpatient treatments. The study also has implications for our therapy setting and program planning. We think about adding a specialized trauma confrontation group, which our patients can enter after completing the afore examined stabilizing group treatment in form of an interval treatment. Patients need to be thoroughly screened for willingness and readiness to undertake exposure treatment.

Further research should include a control group, a higher number of participants and structured interviews to secure diagnoses.

## Conclusion

The current study shows that multimodal, group-based day clinic treatment programs for patients with trauma-related disorders are associated with reductions in depressive and cPTSD symptoms as well as with increases in protective factors like perceived social support and PTG. It also demonstrates that cPTSD patients benefit from such programs and can be successfully treated. On the other hand, our study has identified that the treatment of specific symptoms such as intrusions were undervalued in our concept. We plan to optimize our psychotherapeutic concept according to these results of the study. One optimization possibility would be to consider exposure-based interventions earlier in the therapeutic process.

## Author’s Note

The present work was performed in fulfillment of the requirements for obtaining the degree “Dr. phil” (AP).

## Data Availability Statement

The datasets generated for this study are available on request to the corresponding author.

## Ethics Statement

The studies involving human participants were reviewed and approved by ethics committee of the Medical Faculty of the Friedrich-Alexander University Erlangen-Nürnberg (FAU). The patients/participants provided their written informed consent to participate in this study.

## Author Contributions

AP conceived, designed, conducted, analyzed, and wrote this manuscript. YE, AS, EM, and MS provided feedback and mentorship on each stage of the research design and implementation, including a full review and provision of feedback on the final manuscript.

## Conflict of Interest

The authors declare that the research was conducted in the absence of any commercial or financial relationships that could be construed as a potential conflict of interest.
