# Supplementary material for: Effectiveness of a Multimodal, Day Clinic Group-Based Treatment Program for Trauma-Related Disorders: Differential Therapy Outcome for Complex PTSD vs. Non-Complex Trauma-Related Disorders
Source: Front Psychiatry. 2019 Nov 7;10:800. doi: 10.3389/fpsyt.2019.00800 (PMC6853865; doi:10.3389/fpsyt.2019.00800)
Supplement: Supplementary file 1 [file Table_1.docx]

Table in supplementary material. Comparison of scores for patients with and without complex PTSD: Symptom scores.

| **Variables** | **T1** | | | | | **T2** | | | | |
| --- | --- | --- | --- | --- | --- | --- | --- | --- | --- | --- |
|  | **Non-complex sub-group** | **Complex sub-group** | **Comparison** | | | **Non-complex sub-group** | **Complex sub-group** | **Comparison** | | |
|  | **M (SD)** | **M (SD)** | **t** | **p** | **d** | **M (SD)** | **M (SD)** | **t** | **p** | **d** |
| ETI total | 33.04 (15.47) | 47.92 (14.71) | -3.899 | **<0.001** | 0.982 | 31.52 (17.78) | 50.15 (12.02) | -4.736 | **<0.001** | 1.196 |
| ETI PTSD | 27.34 (11.64) | 37.88 (10.88) | -3.694 | **<0.001** | 0.931 | 26.03 (13.32) | 39.34 (7.83) | -5.030 | **<0.001** | 1.178 |
| ETI intrusion | 8.42 (3.85) | 10.85 (3.89) | -2.489 | **0.016** | 0.628 | 8.28 (4.76) | 11.95 (2.98) | -3.787 | **<0.001** | 0.897 |
| ETI avoidance | 9.93 (4.79) | 15.47 (4.65) | -4.647 | **<0.001** | 1.171 | 9.35 (5.24) | 16.00 (3.46) | -5.997 | **<0.001** | 1.457 |
| ETI hyper-arousal | 8.99 (3.94) | 11.56 (3.51) | -2.715 | **0.009** | 0.683 | 8.40 (4.05) | 11.39 (2.51) | -3.763 | **<0.001** | 0.861 |
| ETI dissociation | 5.69 (4.95) | 10.04 (4.64) | -3.580 | **0.001** | 0.903 | 5.49 (5.30) | 10.81 (4.74) | -4.070 | **<0.001** | 1.050 |
| SkPTBS | 9.40 (8.32) | 69.24 (20.19) | -14.763 | **<0.001** | 4.088 | 19.72 (24.29) | 56.07 (33.62) | -4.843 | **<0.001** | 1.268 |
| BDI-II | 25.65 (10.52) | 37.51 (9.40) | -4.658 | **<0.001** | 1.179 | 21.77 (12.49) | 32.49 (12.15) | -3.640 | **0.001** | 0.868 |
| PHQ somatiza-tion | 12.50 (6.09) | 15.71 (4.32) | -2.424 | **0.018** | 0.594 | 13.52 (6.84) | 18.09 (3.76) | -2.953 | **0.004** | 0.798 |
| PHQ pain | 5.57 (3.46) | 6.65 (2.61) | -1.339 | 0.186 | 0.346 | 6.00 (3.47) | 7.75 (2.57) | -1.786 | 0.079 | 0.562 |
| PHQ other somatiza-tion | 3.63 (3.01) | 5.23 (2.48) | -2.220 | **0.030** | 0.572 | 4.37 (3.18) | 6.95 (2.02) | -3.357 | **0.001** | 0.940 |

ETI = Essen Trauma Inventory; ETI total: sum score of all four subscales; ETI PTSD: sum score calculated on the basis of the three subscales that are relevant for the cut-off for a PTSD diagnosis SkPTBS = Screening for complex PTSD; BDI-II = Beck Depression Inventory-Revision; PHQ = Patient Health Questionnaire; PHQ pain: items asking about experiencing pain; PHQ other somatization: items asking about other somatoform symptoms; varying sample sizes and degrees of freedom (df).
