# Supplementary material for: Effectiveness of a Multimodal, Day Clinic Group-Based Treatment Program for Trauma-Related Disorders: Differential Therapy Outcome for Complex PTSD vs. Non-Complex Trauma-Related Disorders
Source: Front Psychiatry. 2019 Nov 7;10:800. doi: 10.3389/fpsyt.2019.00800 (PMC6853865; doi:10.3389/fpsyt.2019.00800)
Supplement: Supplementary file 2 [file Table_2.docx]

Table in supplementary material. Comparison of scores for patients with and without complex PTSD: Protective factors.

| **Variables** | **T1** | | | | | **T2** | | | | | |
| --- | --- | --- | --- | --- | --- | --- | --- | --- | --- | --- | --- |
|  | **Non-complex sub-group** | **Complex sub-group** | **Comparison** | | | **Non-Complex sub-group** | **Complex sub-group** | **Comparison** | | | |
|  | **M (SD)** | **M (SD)** | **t** | **p** | **d** | **M (SD)** | **M (SD)** | **t** | | **p** | **d** |
| PTGI total | 40.63 (16.37) | 40.40 (19.50) | 0.046 | 0.964 | -0.013 | 45.97 (15.61) | 44.10 (19.04) | 0.863 | | 0.392 | -0.109 |
| PTGI Relating to Others | 15.86 (5.99) | 15.35 (8.00) | 0.330 | 0.743 | -0.074 | 17.69 (6.94) | 15.80 (7.01) | | 1.184 | 0.242 | -0.271 |
| PTGI New Possibilities | 8.07 (4.89) | 9.90 (5.65) | -1.220 | 0.228 | 0.350 | 10.70 (4.50) | 11.10 (5.16) | 0.016 | | 0.987 | 0.083 |
| PTGI Personal Strength | 7.78 (4.79) | 6.70 (4.14) | 0.823 | 0.415 | -0.239 | 10.03 (4.64) | 7.90 (4.39) | 1.735 | | 0.089 | -0.470 |
| PTGI Spiritual Change | 1.74 (2.49) | 2.30 (3.26) | -0.691 | 0.493 | 0.197 | 1.77 (2.63) | 2.70 (3.16) | -0.608 | | 0.546 | 0.324 |
| PTGI Appreciation of Life | 7.13 (3.43) | 6.15 (3.75) | 0.960 | 0.342 | -0.274 | 7.06 (3.45) | 6.60 (3.63) | 0.744 | | 0.461 | -0.130 |
| FSoz-U | 38.21 (13.12) | 27.15 (14.02) | 3.268 | **0.002** | -0.818 | 37.50 (13.85) | 32.00 (13.90) | 1.583 | | 0.118 | -0.396 |

PTGI = Posttraumatic Growth Inventory; FSozU = questionnaire for perceived social support; varying sample sizes and degrees of freedom (df).
